# BCAP31 Alleviates Lipopolysaccharide-Mediated Acute Lung Injury via Induction of PINK1/Parkin in Alveolar Epithelial Type II Cell

**DOI:** 10.34133/research.0498

**Published:** 2024-10-08

**Authors:** Pingjun Zhu, Xi Wang, Qingfeng Wu, Jianbo Zhu, Yifan Que, Yan Wang, Yongkai Ding, Yang Yang, Jie Jin, Xin Zhang, Qian Xu, Qinge Yong, Christopher Chang, Guogang Xu, Yingzhen Du

**Affiliations:** ^1^ Chinese PLA General Hospital, Medical School of Chinese PLA, Beijing 100853, China.; ^2^ The Second Medical Center & National Clinical Research Center for Geriatric Diseases, Chinese PLA General Hospital, Beijing 100853, China.; ^3^ Xianning Medical College, Hubei University of Science and Technology, Xianning, China.; ^4^ Department of Emergency, Beijing Tsinghua Changgung Hospital, Tsinghua University, Beijing 102218, China.; ^5^ Division of Immunology, Allergy and Rheumatology, Joe DiMaggio Children’s Hospital, Memorial Healthcare System, Hollywood, FL 33021, USA.

## Abstract

**Background:** B-cell receptor-associated protein 31 (BCAP31) has protective effects against alveolar epithelial type II cells (AECII) damage by inhibiting mitochondrial injury in acute lung injury (ALI) induced by lipopolysaccharide (LPS), whereas the precise mechanism is still unclear. It is known that PTEN-induced putative kinase 1 (PINK1)/Parkin-mediated mitophagy can remove damaged mitochondria selectively, which may be involved in BCAP31 protection against mitochondrial injury. **Methods:** In the current study, ALI mice models were established by using surfactant protein C (Sftpc)-BCAP31 transgenic mice (BCAP31^TG^ mice) and AECII-specific BCAP31 knockout mice (BCAP31^CKO^ mice) treated with LPS. **Results:** BCAP31 expression in lung tissue and AECII were inhibited in ALI mice. Under LPS challenge, lower level of BCAP31 was found to correlate positively with pathological injury of the lung, respiratory dysfunction, mortality rates, inflammation response, and AECII damage. Further study showed that down-regulation of BCAP31 induced decreased phosphorylation of PINK1 via reduced binding to PINK1, thereby restraining PINK1/Parkin-mediated mitophagy. Down-regulation of mitophagy promoted mitochondrial injury, as shown by the increase in mitochondrial permeability transition pore opening rate, together with enhanced mitochondrial reactive oxygen species (mROS), which were accompanied by increased cellular apoptosis and reactive oxygen species (ROS). The increased cellular ROS contributed to the inflammatory response via activation of nuclear factor κB (NF-κB). In contrast, BCAP31 overexpression promoted phosphorylation of PINK1 and PINK1/Parkin-mediated mitophagy, thus blocking the mROS/ROS/NF-κB pathway, favoring a protective condition that ultimately led to the inhibition of AECII apoptosis and inflammatory response in LPS-induced ALI. **Conclusion:** Ultimately, BCAP31 alleviated ALI by activating PINK1/Parkin-mediated mitophagy and blocking the mROS/ROS/NF-κB pathway in AECII.

## Introduction

Acute respiratory distress syndrome (ARDS) is the most severe clinical manifestation of acute lung injury (ALI). ALI/ARDS are usually featured by respiratory distress and intractable hypoxemia with high morbidity and mortality [1]. Although numerous studies on interventions for ALI have been conducted, the translation of pathophysiologic mechanisms to clinical care remains an ongoing challenge [2]. It is believed that alveolar epithelial type II cells (AECII) play an essential role in lung function and injury repair by affecting lung surface-active substance metabolism, regulation of alveolar liquid, and differentiation of alveolar epithelial type I cells (AECI) [3]. Recent studies confirmed that AECII are also important target cells during lipopolysaccharide (LPS)-induced ALI. Apoptosis of AECII results in decreased alveolar surfactant synthesis, respiratory membrane destruction, and alveolar occlusion [4]. Inhibition of ACTII apoptosis marked improves lung function and reduces mortality of LPS-induced ALI in vivo. Therefore, focusing on the regulatory mechanism of AECII apoptosis may provide novel insights on the management of ALI.

Mitochondria are the main organelles that supply energy in eukaryotic cells and contribute to cellular physiological processes including oxidative stress, calcium overload, signal transduction, cellular proliferation, and apoptosis. Previous evidence indicates that mitochondrial dysfunction in AECIIis strongly associated with the progression of ALI [5,6]. Our study have previously revealed that LPS caused mitochondrial injury, as indicated by dissipation of mitochondrial membrane potential, decreased production of adenosine triphosphate (ATP), as well as the burst of mitochondrial reactive oxygen species (mROS) in LPS-treated AECII [4]. Mitochondrial ROS (mtROS) breakout has been proven to promote mitochondria-associated endoplasmic reticulum (ER) membrane dysfunction in vitro [7]. In vivo, impaired mitochondrial function is closely associated with impaired pulmonary ventilation in LPS-induced ALI [8]. Moreover, delivery of normal mitochondria to AECII through adipose-derived mesenchymal stem cells can significantly mitigate LPS-induced AECII apoptosis and lung injury [9]. However, the upstream regulatory mechanism of mitochondrial damage in AECII remains elusive.

B-cell receptor-associated protein 31 (BCAP31), which is a 28-kDa transmembrane protein, is extensively expressed in ER and mitochondria-associated membranes (MAMs). Recently, a growing number of researchers reported that BCAP31 is important in the pathogenesis of cancers, inflammation in multiple organs, and other diseases [10,11]. Additionally, BCAP31 is essential in mitochondrial function regulation. BCAP31 also interacts with TOM40 at the ER-mitochondrial contact site to exert regulatory function on mitochondrion [12]. Our previous study revealed that BCAP31 expression is significantly down-regulated in ALI, and overexpression of BCAP31 can significantly inhibit LPS-induced mitochondrial damage and AECII apoptosis [13]. However, the underlying mechanism by which BCAP31 alleviates mitochondrial damage in ALI remains unknown.

Mitophagy is a protective mechanism for maintaining mitochondrial function and plays a vital cytoprotective role in different diseases [14]. Among various signaling pathways in mitophagy, PTEN-induced putative kinase 1 (PINK1)/Parkin-mediated mitophagy is considered the most well-understood and typical pathway [15–17]. In hypoxia-induced ALI, inhibiting mitophagy via genetic abolishment of PINK1 significantly increases the inflammatory reaction and oxidative stress in respiratory system [18]. Conversely, in ALI models induced by cecal ligation and puncture, activating PINK1/Parkin-mediated mitophagy in macrophages can significantly inhibit lung tissue damage [19,20]. Furthermore, the mitophagy activation has been proved to reduce lung injury, as indicated by alleviation of inflammation and improvement in lung edema [21]. Given the fact that PINK1/Parkin-mediated mitophagy is essential for maintaining mitochondrial function in ALI, we speculated that BCAP31 may reduce AECII apoptosis and lung injury through activating the PINK1/Parkin mitophagy pathway. By constructing ALI models with LPS in AECII BCAP31-deficient mice (BCAP31^CKO^) and surfactant protein C (Sftpc)-BCAP31^TG^ mice, how BCAP31 alteration affecting on lung injury, oxidative stress and mortality were studied.

## Results

### BCAP31 presents protective effects on ALI mice induced by LPS

**Fig. 1.**
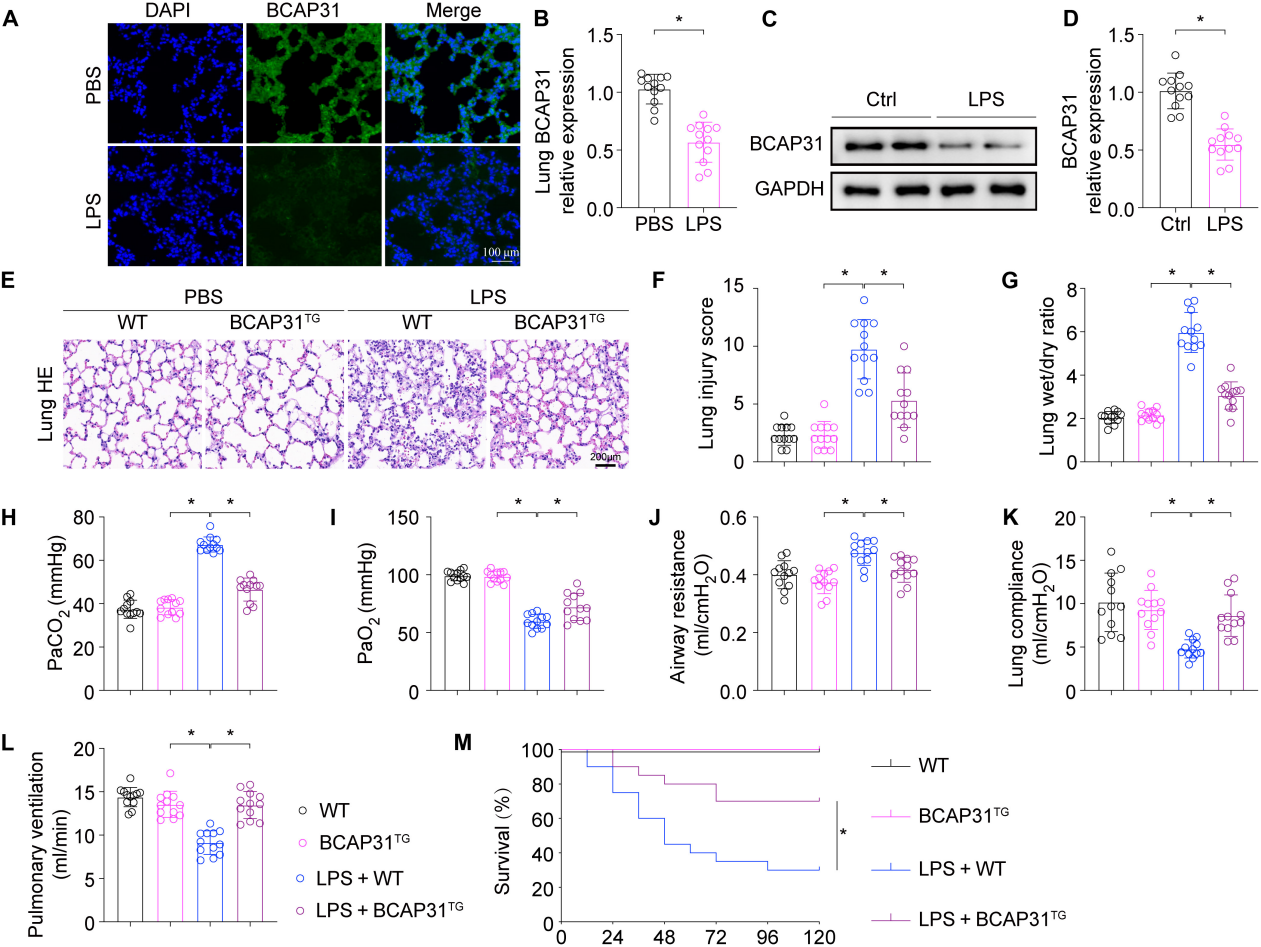
Overexpression of BCAP31 alleviates LPS-mediated ALI (*n* = 12/group). (A and B) The change in BCAP31 expression in lung tissue followed LPS treatment was measured by immunofluorescence staining. (C and D) Primary AECII, isolated from the WT mice, were administrated with LPS. Western blot was applied to measure the change in BCAP31. (E) H&E staining of lung tissue. (F) Lung injury score. (G) Lung W/D ratio. (H and I) Blood gas analysis of the mice with the abdominal aorta blood sampling. (J to L) Respiratory functions referring to airway resistance, lung compliance, and pulmonary ventilation were measured by Buxco. (M) Kaplan–Meier survival curve was used to present percent survival rate (*n* = 20 each group). Mean ± SD was shown in all figures in this research. *P < 0.05. PBS, phosphate-buffered saline; DAPI, 4′,6-diamidino-2-phenylindole; GAPDH, glyceraldehyde-3-phosphate dehydrogenase.

Firstly, we investigated whether the expression of BCAP31 could be changed in the process of ALI pathogenesis. The level of BCAP31 expression was found significantly down-regulated in lung tissue (Fig. 1A and B) following LPS challenge, which was consistent with our previous study. The primary AECII, isolated from the wild-type (WT) mice, were incubated with LPS to imitate ALI in vitro. LPS significantly reduced BCAP31 expression in primary AECII (Fig. 1C and D). To further explore the role of BCAP31 in ALI, Sftpc-BCAP31 transgenic mice (BCAP31^TG^ mice) and AECII-specific BCAP31 knockout mice (BCAP31^CKO^ mice) were produced (Fig. S1A to F). Following treatment with LPS, pathological changes, including inflammatory cell infiltration, diffuse alveolar damage, pulmonary edema, and hemorrhage, were observed under hematoxylin and eosin (H&E) staining. LPS exposure led to marked lung injury (Fig. 1E), while mice with BCAP31 overexpression presented with better histological outcomes after ALI treatment. BCAP31 deletion also significantly aggravated the lung injury caused by LPS (Fig. S1G). Mice in the LPS-treated group presented with a higher wet/dry (W/D) ratio, which is associated with aggravated pulmonary edema, while BCAP31 overexpression alleviated pulmonary edema induced by LPS (Fig. 1F). Interestingly, BCAP31 knockout promoted pulmonary edema in the current mice model (Fig. S1H). Furthermore, the effects of BCAP31 in LPS-induced ALI mice were determined.

Blood gas analysis indicated that LPS treatment was associated with rising level of PaCO_2_ and suppressed PaO_2_ and PaO_2_/FiO_2_ in mice (Fig. 1G to I). Furthermore, LPS was observed to enhance airway resistance (Fig. 1J), companioned with decreased pulmonary compliance and ventilation (Fig. 1K and L). BCAP31 overexpression reduced the LPS-induced respiratory dysfunction. Conversely, BCAP31 deletion aggravated the LPS-induced respiratory dysfunction (Fig. S1I to N). Animal mortality was monitored following LPS treatment. Besides, the survival rates of BCAP31^TG^ mice were significantly higher following ALI than that of WT mice (Fig. 1M). In addition, a lower survival rate was observed in BCAP31^CKO^ mice (Fig. S1O). These results demonstrate that BCAP31 is down-regulated in ALI and that BCAP31 contributes to alleviating the pathological damage of lung tissues, improving pulmonary function, and preventing LPS-related death in mice.

### BCAP31 inhibits LPS-induced AECII damage

**Fig. 2.**
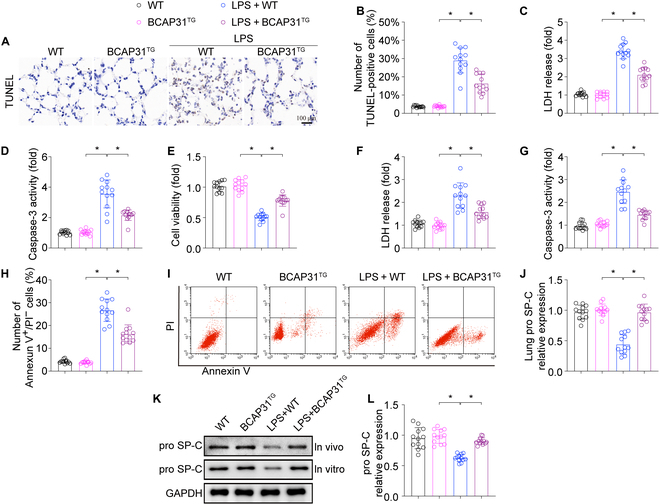
BCAP31 Regulation on alveolar epithelial cell damage in LPS-induced ALI models (*n* = 12/group). (A and B) Percentage of apoptotic alveolar epithelial cells was evaluated by TUNEL staining. (C) Increased LDH release in LPS-induced lung tissue. (D) Caspase-3 activity as a biomarker of apoptosis is significantly increased due to LPS treatment. (E) Cellular viability was detected via MTT assay. (F) Increased LDH release in alveolar epithelial cells after LPS treatment. (G) Caspase-3 activity is significantly increased in alveolar epithelial cells induced by LPS. (H and I) Apoptotic cells was analyzed through flow cytometry with Annexin V/propidium iodide staining. (J to L) Expression of pro SP-C was measured by western blotting. *P < 0.05.

It has been widely acknowledged that AECII damage, including increased apoptosis and cellular secretory dysfunction, is central to the histologic findings of ALI. TUNEL (terminal deoxynucleotidyl transferase-mediated deoxyuridine triphosphate nick end labeling) assay showed that LPS significantly increased the apoptotic cells in lung tissue compared to the WT group (Fig. 2A and B). Furthermore, LPS also induced an increase in lactate dehydrogenase (LDH) release (Fig. 2C) and caspase-3 activity (Fig. 2D). However, the above effect was reduced in BCAP31^TG^ mice. In contrast, BCAP31 deletion promoted LPS-induced cellular apoptosis, indicated by the increased apoptotic cells in lung tissue, LDH release, and caspase-3 activity (Fig. S2A to D). The regulatory function of BCAP31 in LPS-induced ALI is further supported by cellular evidence from mouse AECII. BCAP31 overexpression and BCAP31 knockout had no marked effect on cell viability and apoptosis (Fig. 2E to I and Fig. S2E and F). BCAP31 overexpression significantly enhanced AECII survival under LPS stimulation as indicated by the 3-(4,5-dimethylthiazol-2-yl)-2,5-diphenyltetrazolium bromide (MTT) assay (Fig. 2E), LDH release (Fig. 2F), and caspase-3 activity (Fig. 2G). Annexin V/propidium iodide flow-cytometry for quantitative analysis of cellular apoptosis was achived. As shown in Fig. 2I and J, BCAP31 overexpression attenuated LPS-mediated cellular apoptosis. However, a higher level of cellular apoptosis was observed in BCAP31 knockout AECII (Fig. S2E and F). These data indicate that BCAP31 can significantly inhibit AECII apoptosis in ALI.

Furthermore, the level of pro SP-C, which is a precursor protein of pulmonary surfactant, was measured to assess AECII function via western blot and immunofluorescence staining. LPS treatment turned out to remarkably reduce pro SP-C level in vivo and in vitro comparing with WT group, which was reversed by BCAP31 overexpression (Fig. 2J to L). Besides, BCAP31 knockout mice group presented with significantly higher levels of pro SP-C compared with WT mice (Fig. S2J and K). Similar results were confirmed in vitro (Fig. S2L and M). Overall, these data support that BCAP31 is vital in preventing LPS-induced ALI via inhibition of AECII apoptosis and secretory dysfunction.

### BCAP31 suppresses LPS-induced inflammatory responses via ROS/NF-κB pathway

**Fig. 3.**
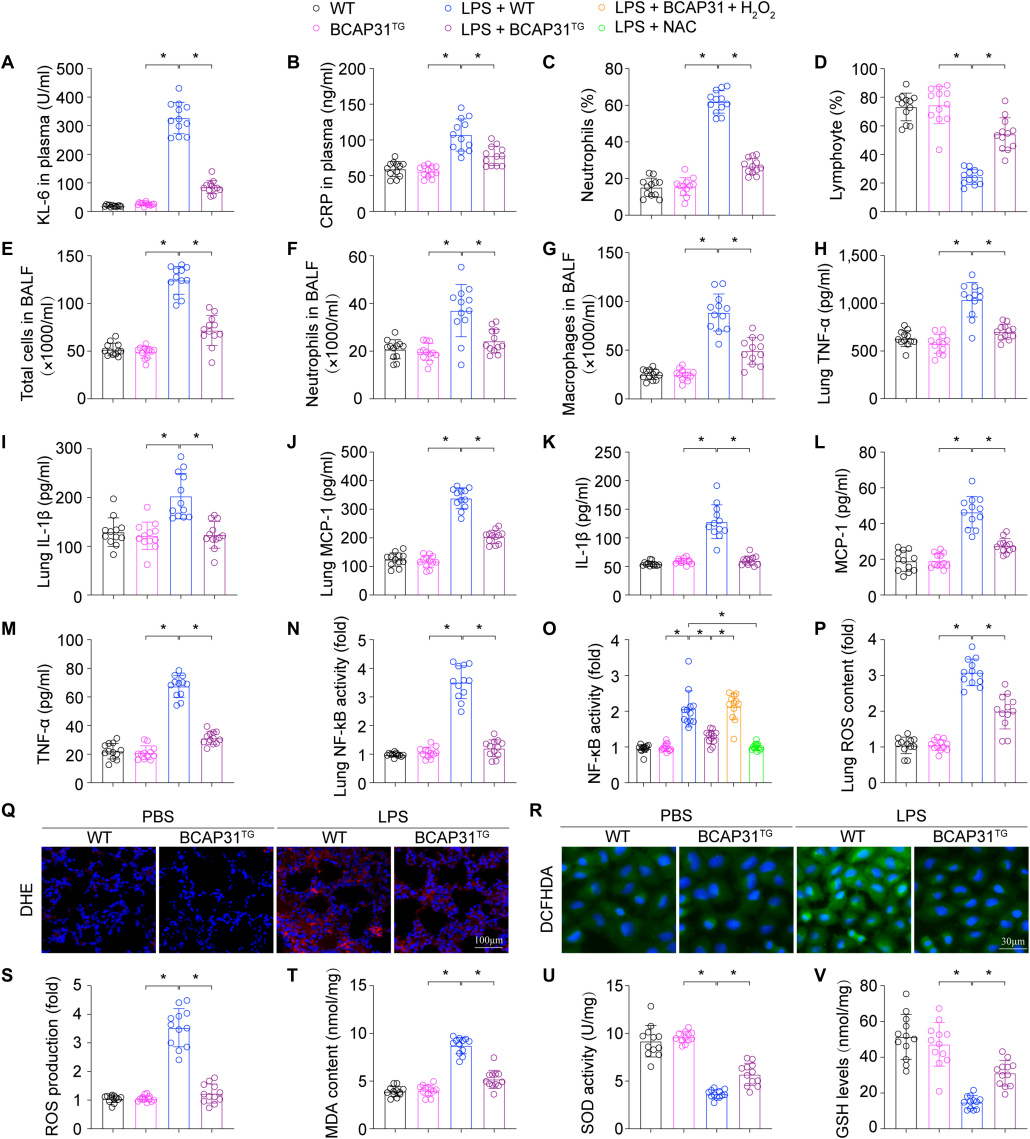
Regulation of BCAP31 on oxidative stress and inflammation response activated by LPS (*n* = 12/group). (A and B) KL-6 and CRP levels in plasma. (C and D) Lymphocytes (%) and neutrophils (%) in blood. BALF total cell (E), neutrophil (F), and macrophage (G) were measured to evaluate LPS-induced injury. (H to J) Concentrations of cytokines including TNF-α, IL-1β, and MCP-1 in lung tissue. (K to M) ELISA of TNF-α, IL-1β, and MCP-1 levels. (N) NF-κB activity in lung was performed via ELISA. (O) NAC was applied to scavenge those excessive ROS due to LPS administration. In contrast, we applied H_2_O_2_, in BCAP31-overexpressed cells, to reverse oxidative stress. The activity of NF-κB in AECII was measured via ELISA in respond to LPS. (P and Q) ROS content in the lung tissue. (R and S) 2,7-Dichlorodi-hydrofluorescein diacetate was performed to evaluate ROS change in AECII. MDA content (T), SOD activity (U), and GSH levels (V) in lung tissue. *P < 0.05.

It is known that inflammatory reactions are essential in the ALI pathophysiology [22,23]. We next investigated the effect of BCAP31 on inflammatory responses under the challenge of ALI. Here, we found that BCAP31 overexpression significantly reduced Krebs von den Lungen-6 (KL-6), C-reactive protein (CRP), and peripheral blood neutrophils (%) (Fig. 3A to C) and increased peripheral blood lymphocytes (%) (Fig. 3D). Besides, reduced total cell (Fig. 3E), neutrophil (Fig. 3F), and macrophage counts (Fig. 3G) in bronchoalveolar lavage fluid (BALF) of BCAP31 overexpression group were observed. BAP31 also increased inflammatory cytokines in the lung (Fig. 3H to J) and AECII (Fig. 3K to M), indicated by tumor necrosis factor-α (TNF-α), interleukin-1β (IL-1β), and monocyte chemoattractant protein-1 (MCP-1). Currently, nuclear factor κB (NF-κB) has been recognized as important part in the inflammatory responses associated with ALI. BCAP31 overexpression reduced NF-κB activity both in vivo (Fig. 3N) and in vitro (Fig. 3O). Compared to the WT group, BCAP31 deletion facilitated inflammatory responses in LPS-mediated ALI, as shown by increases in CRP (Fig. S3A) peripheral blood neutrophils (%) (Fig. S3B), the total cell numbers in BALF (Fig. S3C), and TNF-α in lung tissue (Fig. S3D). Furthermore, BCAP31 deletion enhanced activity of NF-κB in lung tissue (Fig. S3E) and AECII (Fig. S3F) under LPS stimulation. Thus, these findings indicated that BCAP31 alleviates the inflammation reaction by inhibition of NF-κB pathway.

BCAP31 exerts a regulatory function on ROS, which further activates the NF-κB pathway. Thereafter, we also studied the effect of BCAP31 on oxidative stress levels in ALI. LPS treatment significantly enhanced ROS content in vivo (Fig. 3P and Q) and in vitro (Fig. 3R and S). Malondialdehyde (MDA), a factor associated with the degree of tissue peroxidation damage, was also higher after LPS treatment (Fig. 3T). However, the above changes were reversed by BCAP31 overexpression. Overexpression of BCAP31 also restored the reduced activity of superoxide dismutase (SOD) and glutathione (GSH) (Fig. 3U and V) in ALI mice lung tissue. Conversely, BCAP31 knockout enhanced ROS content in vivo and in vitro (Fig. S3G to J). These findings suggest that BCAP31 ameliorates oxidative stress in ALI. To investigate whether higher ROS content was related to an NF-κB-mediated inflammatory response, N-acetylcysteine (NAC) was used as an ROS scavenger to eliminate excessive ROS following LPS induction. We administered H_2_O_2_ in BCAP31-overexpressed cells to reverse oxidative stress. We confirmed by enzyme-linked immunosorbent assay (ELISA) that NAC significantly inhibited NF-κB activity under LPS, similar to the results of BCAP31 overexpression (Fig. 3O). Conversely, H_2_O_2_ reactivated NF-κB in BCAP31-overexpressed cells. Given the evidence above, these results jointly suggest that BCAP31 attenuates LPS-related inflammation reaction via ROS/NF-κB pathway in ALI.

### BCAP31 reduces ROS generation by sustaining mitochondrial integrity

**Fig. 4.**
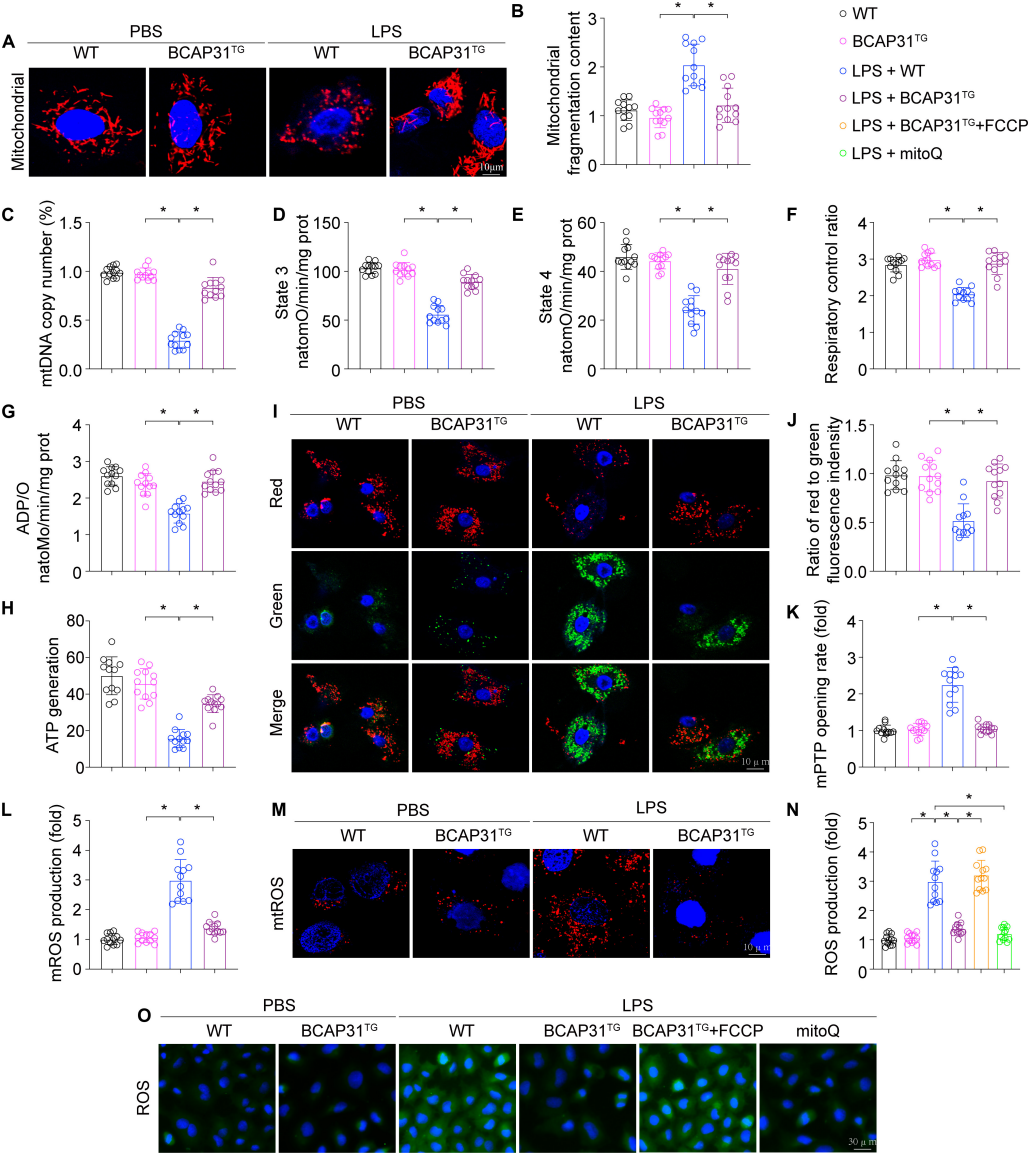
BCAP31 reverses LPS-induced oxidative stress via inhibiting mitochondrial dysfunction and mtROS (*n* = 12/group). (A and B) AECII mitochondria was labeled with anti-Tom20 antibody to evaluate mitochondria fragmentation. (C) The change of mtDNA copy number. (D to G) Effect of BCAP31 on respiratory chains referring to state 3 respiration, state 4 respiration, respiratory control ratio (RCR [state 3/state 4]) and ADP/O. (H) Determination of ATP generation associated with mitochondrial dysfunction. (I and J) JC-1 staining was used to determine mitochondrial membrane potential. (K) mPTP opening rate. (L and M) Mitochondrial ROS contents and (N and O) cellular ROS production were measured by immunofluorescence staining. *P < 0.05.

Our previous studies confirmed that the mitochondria are major contributors to cellular oxidative stress [24,25]. To determine the underlying mechanism of oxidative stress by BCAP31 in ALI, we focused on mitochondrial injury. Firstly, changes of mitochondrial morphology associated with LPS that were observed in our study included extensive round mitochondrial fragments of different sizes (Fig. 4A and B). Moreover, mitochondrial DNA (mtDNA) replication was down-regulated upon LPS stimulation, but BCAP31 overexpression reversed those changes (Fig. 4A to C). Electron transport chain complexes are mostly encoded and regulated by mtDNA. We determined that electron transport chain complex-dependent mitochondrial physiological processes, including the state 3/4 respiratory rate, the respiratory rate, ATP synthesis (ADP/O), and ATP generation efficiency, were greatly inhibited under LPS challenge (Fig. 4D to H). Moreover, LPS also contributed to dissipative mitochondrial membrane potential and further increased mitochondrial permeability transition pore (mPTP) opening rate (Fig. 4I to K). Again, these changes were reversed by BCAP31 overexpression, which were coupled with decreased mtROS (Fig. 4L and M). Because of mPTP opening rate enhancement, mtROS can be released from the injured mitochondria into cytoplasm, which further exacerbated cellular oxidative injury. Subsequently, we used mitoQ, a mtROS scavenger, as the positive control group. Carbonyl cyanide-p-trifluoromethoxyphenylhydrazone, a mitochondrial respiration uncoupler, was used in BCAP31-overexpressed cells to reverse mROS. Notably, application of mitoQ reduced cellular ROS under LPS stress, similar to the results of BCAP31overexpression (Fig. 4N and O). In contrast, reactivation of mtROS by carbonyl cyanide-p-trifluoromethoxyphenylhydrazone ablated the antioxidative effect of BCAP31. These results demonstrate that BCAP31 is involved in ameliorating LPS-mediated oxidative stress by reducing mROS.

### BCAP31 inhibits mitochondrial damage and cellular apoptosis via mitophagy activation

**Fig. 5.**
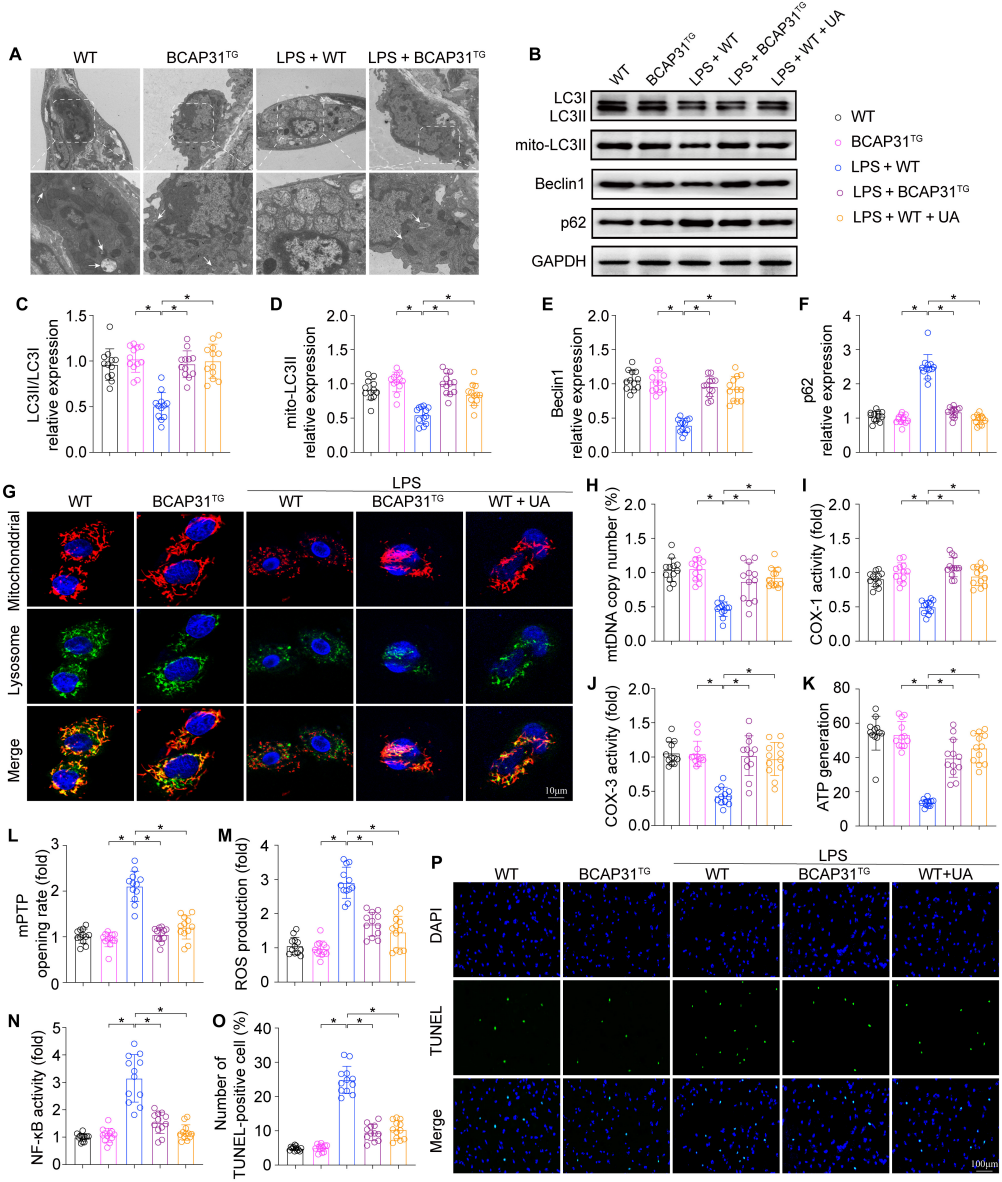
BCAP31 protects against ALI by promoting mitophagy (*n* = 12/group). (A) Representative transmission electron microscopy (TEM) images of double membrane autophagosome formation (white arrow) in mice AECII in vivo. (B to F) Levels of LC3II/LC3I, mito-LC3II, Beclin 1, and p62 were measured by Western blot. (G) LPS significantly reduced mitophagy, as indicated by less mitochondria or fragmented mitochondria in the lysosome. (H) Change in mtDNA copy number. (I and J) The activity of the mitochondrial respiratory complexes was evaluated by ELISA. (K) ATP generation. (L) mPTP opeining rate. (M) Cellular ROS content was determined by immunofluorescence assay. (N) The change in NF-κB activity in AECII. (O and P) TUNEL fluorescence images in vitro. *P < 0.05.

To investigate the underlying mechanism by which BCAP31 inhibits mitochondrial damage, we examined mitophagy. First, we performed transmission electron microscopy analysis to determine the degree of mitophagy in ALI models in vivo. Besides, mitochondria in AECII turned to be smaller and punctate under LPS treatment. LPS also significantly reduced mitophagy, as indicated by less mitochondria or fragmented mitochondria (Fig. 5A). In contrast, BCAP31 overexpression promoted mitophagy. Ursolic acid (UA), a mitophagy activator, was used in BCAP31-overexpressed AECII. The levels of mitophagy biomarkers, including LC3II/LC3I, mito-LC3II, Atg5, Beclin 1, and p62, were measured after LPS stimulation in vitro. LPS significantly reduced the expressions of LC3II/LC3I, mito-LC3II, Atg5, and Beclin 1 but enhanced p62 expression in the AECII (Fig. 5B to F). However, these changes were reversed by BCAP31 overexpression.

Mitophagy was detected with immunofluorescence through colocalization of mitochondria and lysosomes. After LPS stimulation, BCAP31 overexpression promoted association between the mitochondria and lysosome, similar to the UA-treated group (Fig. 5G). Moreover, BCAP31 overexpression and UA inhibited mitochondrial injury, as indicated by increased mtDNA copy number (Fig. 5H), cytochrome c oxidase subunit I/III (COX-I/III) activity (Fig. 5I and J), ATP generation (Fig. 5K) and decreased mPTP opening rate (Fig. 5L). As shown in Fig. 5M and N, UA treatment inhibited ROS generation and NF-κB activity after LPS stimulation, which was similar to BCAP31 overexpression. UA also reduced cellular apoptosis as shown by decreased TUNEL^+^ cells (Fig. 5O and P) in vitro. These findings together indicate that BCAP31 inhibits mitochondrial damage by activating mitophagy in LPS-induced ALI.

### BCAP31 promotes mitophagy by increasing the phosphorylation of PINK1

**Fig. 6.**
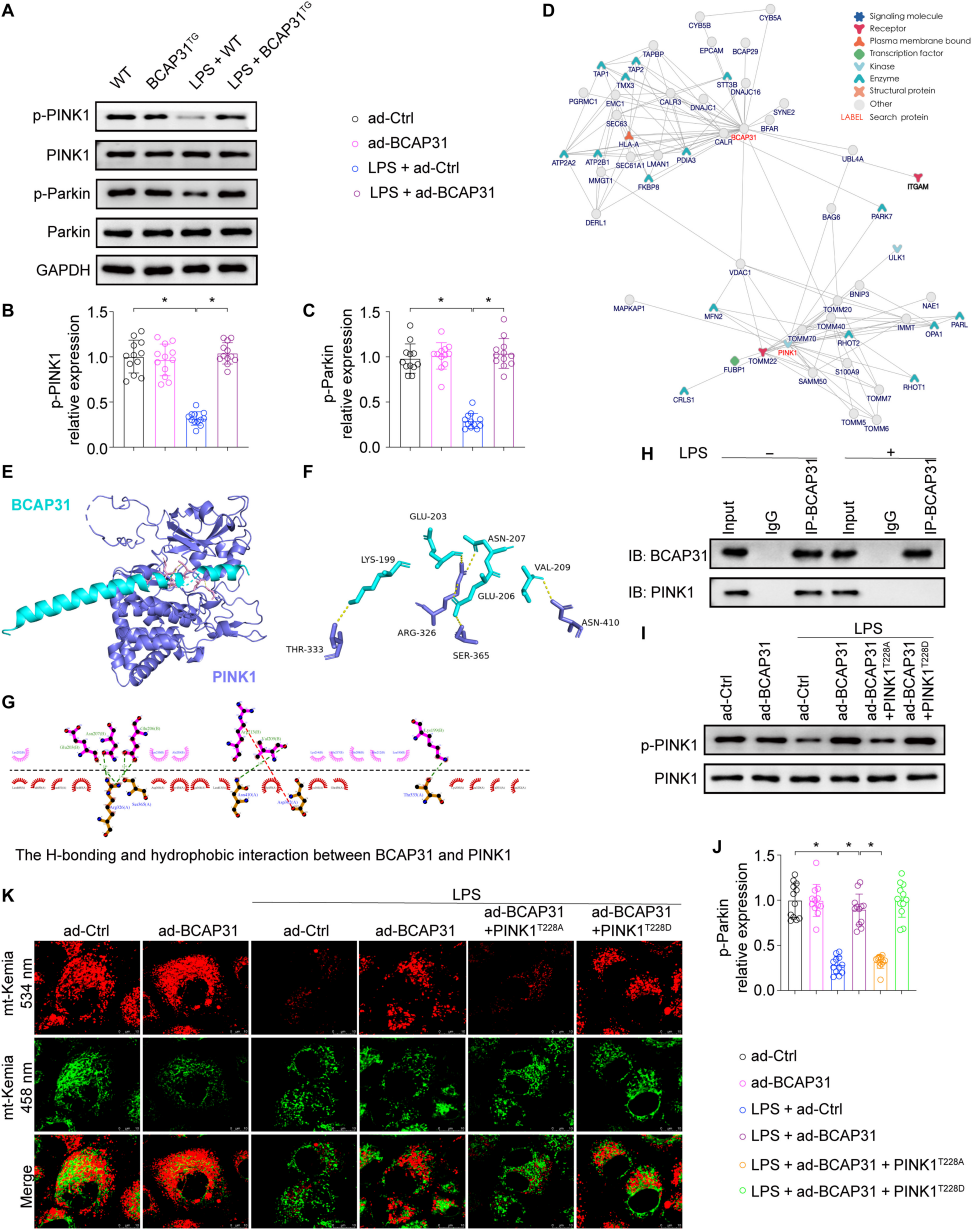
BCAP31 evokes mitophagy via binding PINK1 and promoting PINK1 phosphorylation (*n* = 12/group). (A to C) Expression of p-PINK1 and p-Parkin was detected by Western blotting. (D) Predicted interaction between BCAP31 and PINK1, based on inBio Discover analysis. (E to G) Computational docking analysis of BCAP31-PINK1 binding. H-bonding and hydrophobic interactions between BCAP31 and the active region of PINK1 are shown. (H) Co-IP analysis of the interaction between BCAP31 and PINK1. (I and J) Western blot analysis of p-Parkin expression in A549 cells expressing phosphodefective (PINK1T228A) or phosphomimetic (PINK1T228D) KEAP1 mutant proteins. (K) Mitophagy activity was measured with the mt-Keima assay. Increased mitophagic flux in A549 cells was indicated by highlighted yellow signal. *P < 0.05.

The PINK1/Parkin-mediated mitophagy is proved to be an important pathway in the progression of ALI. Phosphorylation of PINK1 at Ser228, which is essential for the recruitment of Parkin in mitochondrial damage, leads to activation of mitophagy. The PINK1/Parkin pathway was investigated to further unveil the detailed mechanism of BCAP31-mediated mitophagy activation. Western blots showed that LPS inhibited PINK1 and Parkin phosphorylation in AECII, which was reversed by up-regulated BCAP31 (Fig. 6A to C). Considering the role of BCAP31 and PINK1 in MAMs, we hypothesized that BCAP31 directly binds to PINK1 and promotes its phosphorylation.

inBio Discover analysis (https://inbio-discover.com) was used for prediction of the interaction between BCAP31 and PINK1 (Fig. 6D), and molecular docking analysis was then used for subsequent verification (Fig. 6E to G), which provided evidence that BCAP31 interacts with PINK1 via H-bonding and hydrophobic interactions. Thereafter, results of coimmunoprecipitation assay indicated that LPS treatment reduced the interaction between BCAP31 and PINK1 (Fig. 6H). To confirm whether BCAP31 activates mitophagy via phosphorylated PINK1, we then transfected phosphodefective and phosphomimetic PINK1 variant proteins (PINK1^T228A^ and PINK1^T228D^) into Ad-BCAP31-expressing A549 cells alternatively. Under LPS challenge, PINK1^T228A^ transfection prevented phospho-Parkin expression (Fig. 6I and J) and mitophagy, as indicated by the mt-Kemia assay (Fig. 6K), whereas those effects were abrogated by PINK1^T228D^ transfection. These results jointly demonstrate that BCAP31 exerts regulatory effect on mitophagy by promoting the phosphorylation of PINK1.

### BCAP31 overexpression prevents from mitochondrial damage and cellular apoptosis via PINK1 phosphorylation in vitro

**Fig. 7.**
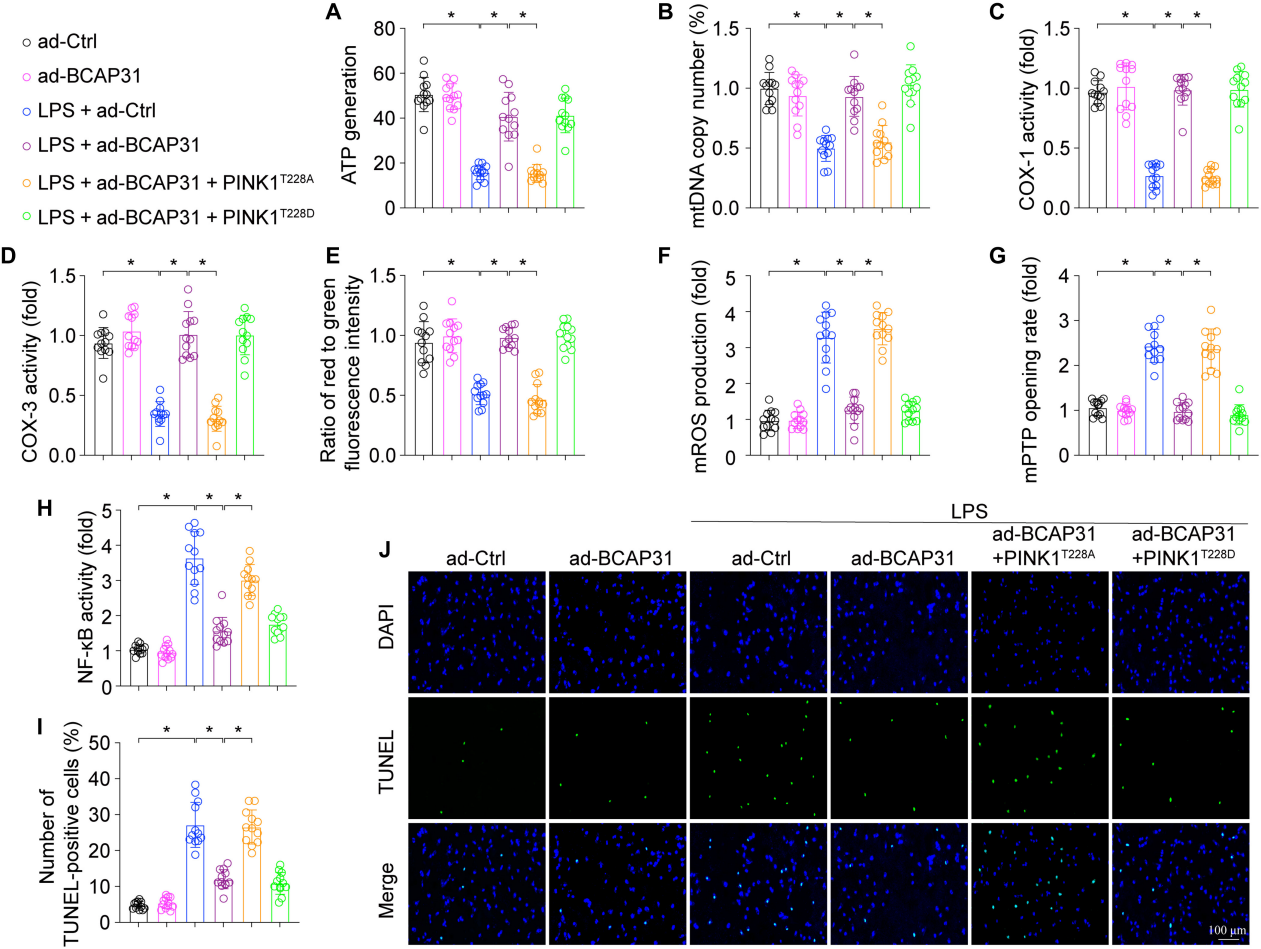
BCAP31 inhibits LPS-induced mitochondrial injury and cellular apoptosis via PINK1 phosphorylation (*n* = 12/group). (A) Determination of ATP generation by ELISA. (B) The mtDNA copy numbers were measured by RT-PCR. (C and D) Changes in COX-I and COX-III activity. (E) JC-1 staining showing mitochondrial membrane potential. (F) Production of mtROS through immunofluorescence staining. (G) Change in mPTP opening. (H) NF-κB activity. (I and J) TUNEL staining was used to assess the apoptotic rate of alveolar epithelial cells. *P < 0.05.

We examined whether phosphorylation of PINK1 induces a protective effect of BCAP31 overexpression on mitochondrial and alveolar cell damage caused by LPS exposure. Mitochondrial function was evaluated in A549 cells cotransfected with Ad-BCAP31 and PINK1^T228A^. PINK1^T228A^ expression eliminated the protective effects of BCAP31 overexpression on mitochondrial ATP generation (Fig. 7A), the activity of mitochondrial respiratory chain complexes (Fig. 7B to D), mitochondrial membrane potentials (Fig. 7E), mROS generation (Fig. 7F), and mPTP opening rate (Fig. 7G). Furthermore, PINK1^T228A^ restored NF-κB activity (Fig. 7H) and promoted cellular apoptosis in BCAP31-overexpressed A549 cells. These results show that BCAP31 inhibits LPS-induced mitochondrial injury and alveolar cell damage through PINK1 phosphorylation.

### Inhibiting PINK1/Parkin-mediated mitophagy weakens the protective effect of BCAP31 in ALI in vivo

**Fig. 8.**
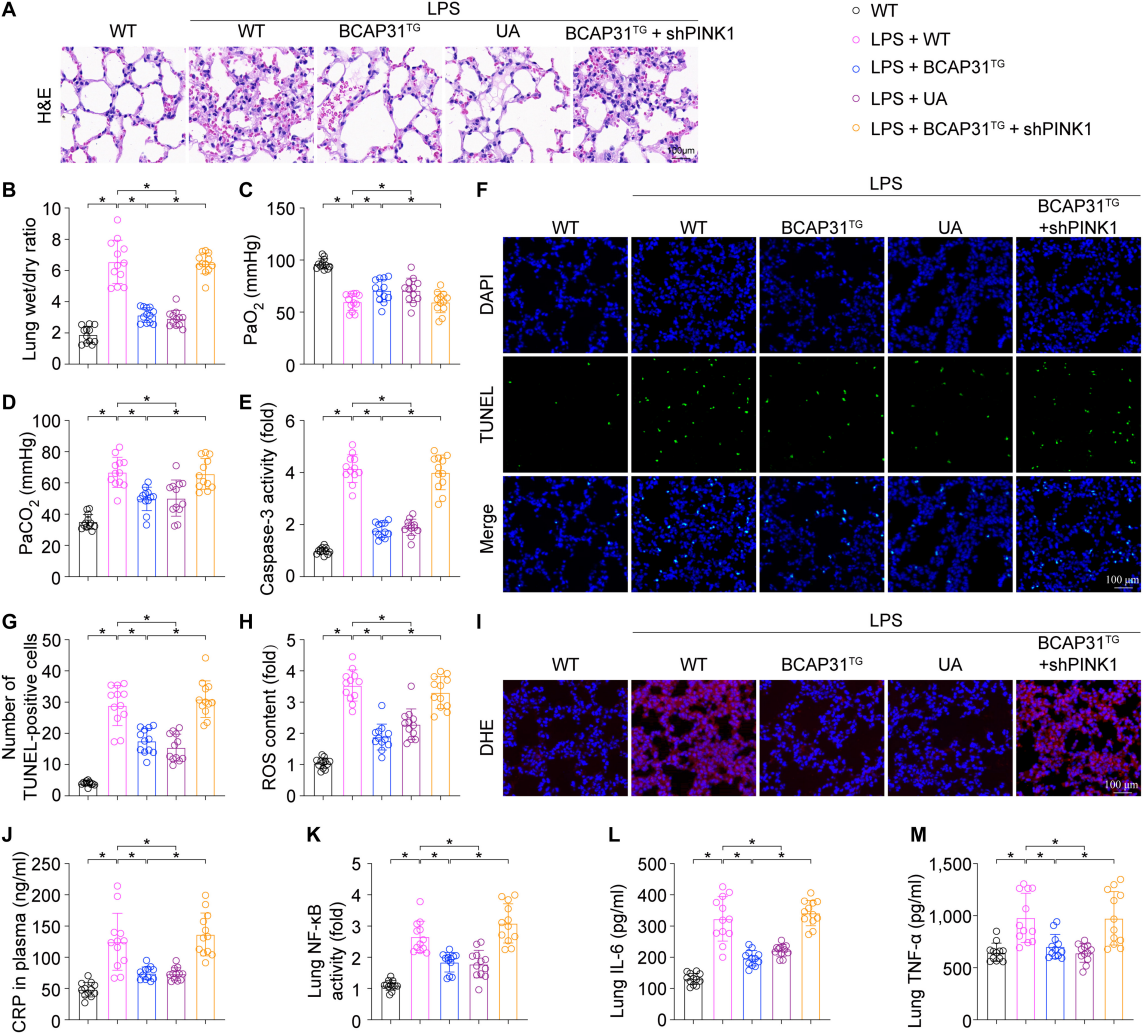
Inhibiting PINK1/Parkin-mediated mitophagy weakens BCAP31 protection on ALI (*n* = 12/group). (A) H&E staining to evaluate the inflammation of lung tissue. (B) Lung wet/dry ratio was measured to evaluate the edema in the lungs. (C and D) Change in blood gases. (E) Caspase-3 level was determined by ELISA. (F and G) Number of apoptotic alveolar epithelial cells was detected by TUNEL staining. (H and I) Lung tissue ROS content. (J) CRP levels in plasma. (K) Lung NF-κB activity was measured by ELISA. (L and M) Expression of IL-6 and TNF-α evaluated by ELISA. *P < 0.05.

To strengthen and confirm our data on BCAP31-mediated mitophagy activation via PINK1/Parkin pathway in vivo, we constructed PINK1 short hairpin RNA (shRNA) (shPINK1) to inhibit PINK1/Parkin-mediated mitophagy in BAP31^TG^ mice, and Urolithin A (UA) was used in WT mice to reactivate PINK1/Parkin-mediated mitophagy under the LPS condition. UA treatment significantly ameliorated the pathological alterations (Fig. 8A), W/D ratio (Fig. 8B) and promoted respiratory function (Fig. 8C and D). Furthermore, treatment with UA inhibited cellular apoptosis (Fig. 8E to G), oxidative stress (Fig. 8H and I), and inflammatory responses (Fig. 8J to M), which is consistent with the results of BCAP31 overexpression. However, the shPINK1 injection abolished the protective effect of BCAP31 overexpression in ALI. These data together indicate that BCAP31 activates PINK1/Parkin-mediated mitophagy to prevent from LPS-induced ALI.

In summary, our study found that BAP31^TG^ mice demonstrated marked pathological alterations in the lung and alleviated inflammatory reactions and oxidative stress, as well as lowered mortality compared to WT mice. Along the same lines, genetic abolishment of BACP31 increased lung injury and death in mice followed LPS challenge. Mechanistically, BCAP31 alleviates AECII damage through activation of the PINK1/mtROS/ROS/NF-κB pathway.

## Discussion

ALI and ARDS, common respiratory diseases with high mortality, are usually characterized by an uncontrolled inflammatory response and AECII apoptosis. Our previous study confirmed that BCAP31 can inhibit LPS-induced ALI via reduced AECII mitochondrial damage and the associated apoptosis. Nonetheless, how BCAP31 inhibits mitochondrial damage in AECII under LPS stress is still unclear. PINK1/Parkin-related mitophagy is regarded as an important part in mitochondrial injury, being involved in the progression of ALI. Therefore, we investigated whether BCAP31 could protect against ALI by activating mitophagy. Our findings showed that BCAP31 overexpression significantly inhibits LPS-induced ALI in vivo. Mechanistically, BCAP31 diminishes mitochondrial injury and apoptosis induced by LPS in AECII through the PINK1/Parkin-mediated mitophagy pathway. To our knowledge, this study firstly elucidates that BCAP31 exerts regulatory effect on LPS-induced ALI via PINK1/Parkin-related mitophagy.

Mitochondrial injury is commonly observed under LPS-induced oxidative stress. In the pathophysiology of sepsis-acute kidney injury, LPS triggers mitochondrial dysfunction, indicated by increased oxidative stress and oxidation of mtDNA [26]. Injury of mtDNA causes reduced activity of respiratory chain complex in mitochondrion and inhibited ATP generation and activation of ROS [9]. The mtROS burst prolongs the mPTP opening time, resulting in leakage of mitochondrial contents and activation of the mitochondria-dependent apoptosis [27]. We also observed that LPS causes AECII mitochondrial damage, as is shown in our study with decreased ATP generation, mitochondrial respiratory chain complex activity, ROS outbreak, mitochondrial membrane potential dissipation, and increased mPTP opening, which are in accordance with our previous findings.

BCAP31, an important component of MAMs, has been shown to regulate the mitochondrial function and ER stress. A previous study confirmed that BCAP31, together with TOM40 at MAMs, enables COX-I components NDUFS4 and NDUFB11 to transfer into mitochondria, suggesting that BCAP31 is highly associated with mitochondrial function [12]. Our preliminary study also found that BCAP31 down-regulation induced by LPS was associated with mitochondrial injury in AECII, although the precise mechanism remained to be elucidated. Herein, we revealed that BCAP31 can inhibit mitochondrial damage and lung injury by promoting mitophagy. The present study fills this theoretical gap.

Mitophagy plays an essential part in maintaining structural and functional mitochondrial homeostasis by selectively clearing damaged mitochondria. Extensive research has been performed and reported on mitophagy in LPS-induced ALI models. For example, the up-regulated peroxisome proliferator-activated receptor gamma coactivator 1-alpha enhanced mitophagy to alleviate ALI in primary AECII apoptosis [28]. Activating mitophagy in alveolar macrophages reduced inflammatory infiltration and oxidative stress in lung tissue after LPS treatment. Many specific receptors, including Fundc1, Bnip3, and PINK1/Parkin, have been reported to activate mitophagy. Our result confirmed that activating PINK1/Parkin-dependent mitophagy is accompanied by decreased mitochondrial injury and AECII apoptosis. Whether other mitophagy regulatory pathways are involved in ALI still needs further exploration.

In addition to mitophagy, mitochondrial fission and fusion are also vital factors in the regulation of mitochondrial function [29]. Our team showed that mitochondrial fusion mediated by OPA1 could be also responsible for mitochondrial damage in AEC [4]. The morphology of alveolar epithelial cells changed from long strips to dots after LPS treatment, as observed by transmission electron microscopy, which was reversed by BCAP31 overexpression. However, it remains unclear whether BCAP31 can mitigate mitochondrial damage by affecting mitochondrial fusion.

Previous studies have shown that BCAP31 can promote the phosphorylation of various proteins, including Akt, Zap70, and epidermal growth factor receptor [30,31]. BCAP31 has been implicated in the modulation of protein phosphorylation through 2 primary mechanisms [11]. Firstly, BAP31 regulates the phosphorylation status of proteins by activating signaling pathways such as Akt and AMP-activated protein kinase. In T cells, BCAP31 supports survival, proliferation, and activation by promoting phosphorylation of key members of T-cell receptor signaling, including glycogen synthase kinase/c-Jun N-terminal kinase/extracellular signal-regulated kinase via AKT pathways [31]. Secondly, BCAP31 facilitates the autophosphorylation of proteins by binding to them. In triple-negative breast cancer cell lines, BCAP31 was found to bind to epidermal growth factor receptor and sustaining its autophosphorylation [30]. In this study, we demonstrated that BCAP31 promotes phosphorylated expression of PINK1 through binding to it, which was consistent with previous study. It should be noted that other factors such as AKT pathway have also been shown to affect PINK1 phosphorylation [32].

PINK1 is normally cleaved by proteases in mitochondrion and degraded by proteasome [33,34]. Autophosphorylation of PINK1 at the Ser228 site has been regarded as essential part for Parkin recruitment into damaged mitochondria [35]. Therefore, we speculate that BCAP31 promotes the phosphorylation level of PINK1 on the one hand by binding to PINK1 to reduce its import into mitochondria for degradation and on the other hand by binding to PINK1 to promote its autophosphorylation.

In conclusion, this study confirms that BCAP31 protects against LPS-related ALI via activation of PINK1/Parkin-mediated mitophagy. Therefore, BCAP31, as a newly discovered positive regulator of mitophagy, can be a promising candidate molecule for developing new approaches against ALI.

## Materials and Methods

### Animals

Animals procedures were authorized by Chinese PLA General Hospital Institutional Animal Care and Use Committee, Beijing. All procedures were performed in compliance with the Guidelines for the Care and Use of Laboratory Animals. BCAP31^flox/flox^ mice were generated as described previously [36] and were crossed with Sftpc-CreERT2 mice to generate AECII BCAP31-deficient mice (BCAP31^CKO^). Male mice of 8 weeks old were intraperitoneally injected with tamoxifen (10 mg/kg) for 5 consecutive days before experiments. Sftpc-BCAP31 transgenic mice (BCAP31^TG^) were achieved in the current study with a protocol previously described. Full-length coding region was inserted into a transgenic targeting cassette with Sftpc gene 5′ flanking region of human. Mice were kept in environment with temperature of 21 ± 2 °C and light/dark cycle of 12 h/12 h, freely accessible food and water, and a relative humidity of 40 to 60%. ALI models (8-wk-old male mice) were constructed by intratracheally administrated LPS (5 mg/kg, Sigma-Aldrich, USA) [37]. PINK1 shRNA or scrambled shRNA (shRNA: 5 mg/kg) was intravenously injected into mice 3 d before treatment for subsequent in vivo experiments [38]. Mice were sacrificed 24 h following LPS treatment.

### Histological analysis

All lung tissues from mice model were extracted and then plunged in 4% paraformaldehyde fixation for 48 h. Subsequently, after being embedded and sliced, H&E staining was applied to evaluate histopathology as previously reported [37].

### BALF analysis

Following the sacrifice of mice, BALF was immediately harvested through intratracheally injected phosphate buffer saline. The protein concentrations in BALF were determined with Bradford Protein Quantification Kit [13]. Wright–Giemsa staining was performed for total cells counting, and hemocytometer was applied to quantitatively calculated total counts of leukocytes, neutrophils, and macrophages [39].

### Lung W/D ratio determination

The lung W/D ratio positively correlated with the degree of pulmonary edema. As previously described, the left lung samples were collected and flushed until no residual blood was left and then were transferred into conditions of 80 °C for 4 d, during which the samples were weighted until reaching a constant mass corresponding to the dry weight.

### Pulmonary function determination and blood gas analysis

After being anesthetized and endotracheally intubated, pulmonary function testing was performed via Buxco pulmonary function (Buxco, Sharon, Connecticut, CT, USA) in mice according to previous study [40]. PaO_2_ and PaCO_2_ in arterial blood were determined with the blood gas analyzer (ABL8000; Radiometer Copenhagen, Denmark) [41].

### LDH release, cell viability, and cytokine determination

LDH release kit (Beyotime, Beijing, China) and the MTT assay were utilized according to the manufacturer’s instructions [42]. ELISA kits (R&D Systems, Minneapolis, MN, USA) were used to evaluate expression of proinflammatory cytokines in BALF, indicated by TNF-α, IL-1β, and MCP-1 [39].

### Cell culture and treatment

The primary AECII were separated from mice according to our previous study. After the sacrifice, lung samples were extracted and incubated in Dispase II (Sigma) to isolate AECII. Subsequently, the isolated AECII were centrifuged and then cultured in DMEM (HyClone, USA) with 10% fetal bovine serum (HyClone, USA) at circumstance of 37 °C and 5% CO_2_, and A549 were incubated in DMEM with 10% fetal bovine serum (HyClone, USA) at circumstance of 37 °C and 5% CO_2_. The cells were incubated with LPS (1 mol/ml) for 24 h to construct the ALI model in vitro [13].

### Cellular apoptosis

As previously reported [42], apoptotic cells were detected with FITC Annexin V Apoptosis Detection Kit (556547, BD Bioscience). Caspase-3 activity was assessed by using the caspase-3 colorimetric assay kit (Millipore, APT165) [43]. TUNEL kit (Keygen, Nanjing, Jiangsu) was used to measure the level of apoptosis [24]. All above data were scanned and recorded with the Olympus fluorescence microscope (BX53, Japan).

### Western blot

Tissue lysis was done by using RIPA Lysis Buffer (Thermo Fisher, China). Equal amounts of proteins were isolated respectively using 10% sodium dodecyl sulfate-polyacrylamide gel electrophoresis. The membranes were then blocked with 5% milk dissolved in tris-buffered saline with Tween 20 and incubated with primary antibodies overnight at 4 °C and then with the secondary antibodies at room temperature for 1 h. All bands were developed and visualized with the enhanced chemiluminescence western blotting kit (Abcam, Cambridge, MA, USA) [25].

### ELISA 

Expressions of KL-6, CRP, TNF-α, IL-1β, MCP-1, NF-κB activity, and caspase-3 activity were determined with commercial ELISA kits (R&D Systems, USA).

### Mitochondrial potential analysis and mPTP opening measurement

Mitochondrial potential was detected by JC-1 staining (T131054, Aladdin, Shanghai, China) [44]. JC-1 (600 nM) was mixed with the medium and incubated for 20 min at 37 °C in darkness for mitochondria staining. After disposing the JC-1 working solution and cell flushing, the images were scanned and obtained with an Olympus BX53 fluorescence microscope. The mPTP opening was determined through tetramethylrhodamine ethyl ester fluorescence [45].

### Mitochondrial morphology, mtDNA copy number, and respiratory chain complex activities

TOM20 primary antibody was applied to label the mitochondria, and change in mitochondrial morphology was recorded via confocal microscopy [4]. Copy number of mtDNA and respiratory chain complex activity assays were performed according to our previous study [24].

### mtROS, ROS analysis, and ATP measurement

mtROS and ATP were measured according to previous study [46]. The AECII were labeled with Mito-SOX Red (Invitrogen, USA) (2.5 μM) to measure mtROS. ROS contents were determined via dihydroethidium (Invitrogen, San Diego, CA, USA) staining and 2,7-dichlorodi-hydrofluorescein diacetate (Beyotime Institute of Biotechnology, Jiangsu, China). MDA content, SOD activity, and GSH level were evaluated with commercial kits (Beyotime Institute of Biotechnology, China). The intensity of fluorescence was detected with a confocal microscope (TCS SP8; Leica, Germany). ATP was measured with an ATP assay kit (Beyotime, China) [42].

### Transmission electron microscopy

The lung sample was firstly fixed with 2.5% glutaraldehyde and flushed with phosphate-buffered saline. Osmium tetroxide (1%) was used for secondary fixation, and then the samples were dehydrated through ethanol with different concentration gradients. Tissues were then infused in varying concentration gradients of epoxy propane soaking and embedding solution. Leica UC7 microtome (Leica, Wetzlar, Germany) was then used to prepare ultrathin slices (100 nm) and stained with uranium dioxide acetate and lead citrate separately. Autophagosomes were then scanned and photographed with a transmission electron microscope (Nippon Electronics Co., Japan) [42,47].

### Docking analysis

The 3-dimensional structures of BCAP31 and PINK1 were acquired from the AlphaFold Protein Structure Database. To predict potential binding modes between BCAP31 and PINK1, Autodock Vina (version 1.1.2) was employed. The most probable binding mode was determined based on the docking solution with the lowest binding free energy, as previously described in our study [48].

### Statistical analysis

One-way analysis of variance was used in analyzing all the data including in the current study. Numbers were represented as mean ± SD. Statistical Package for the Social Sciences (SPSS, Inc., Chicago, IL, USA) software, version 20.0, was applied for data analysis, and *P* < 0.05 was regarded as statistically marked.

## Data Availability

The original data presented in the study are included in the article; further inquiries can be directed to the corresponding author/s.
