# Supplementary material for: BCAP31 Alleviates Lipopolysaccharide-Mediated Acute Lung Injury via Induction of PINK1/Parkin in Alveolar Epithelial Type II Cell
Source: Research (Wash D C). 2024 Oct 8;7:0498. doi: 10.34133/research.0498 (PMC11458857; doi:10.34133/research.0498)
Supplement: Supplementary 1 — Figs. S1 to S3 [file research.0498.f1.pdf]

Supplemental Figures

Supplementary Figure1

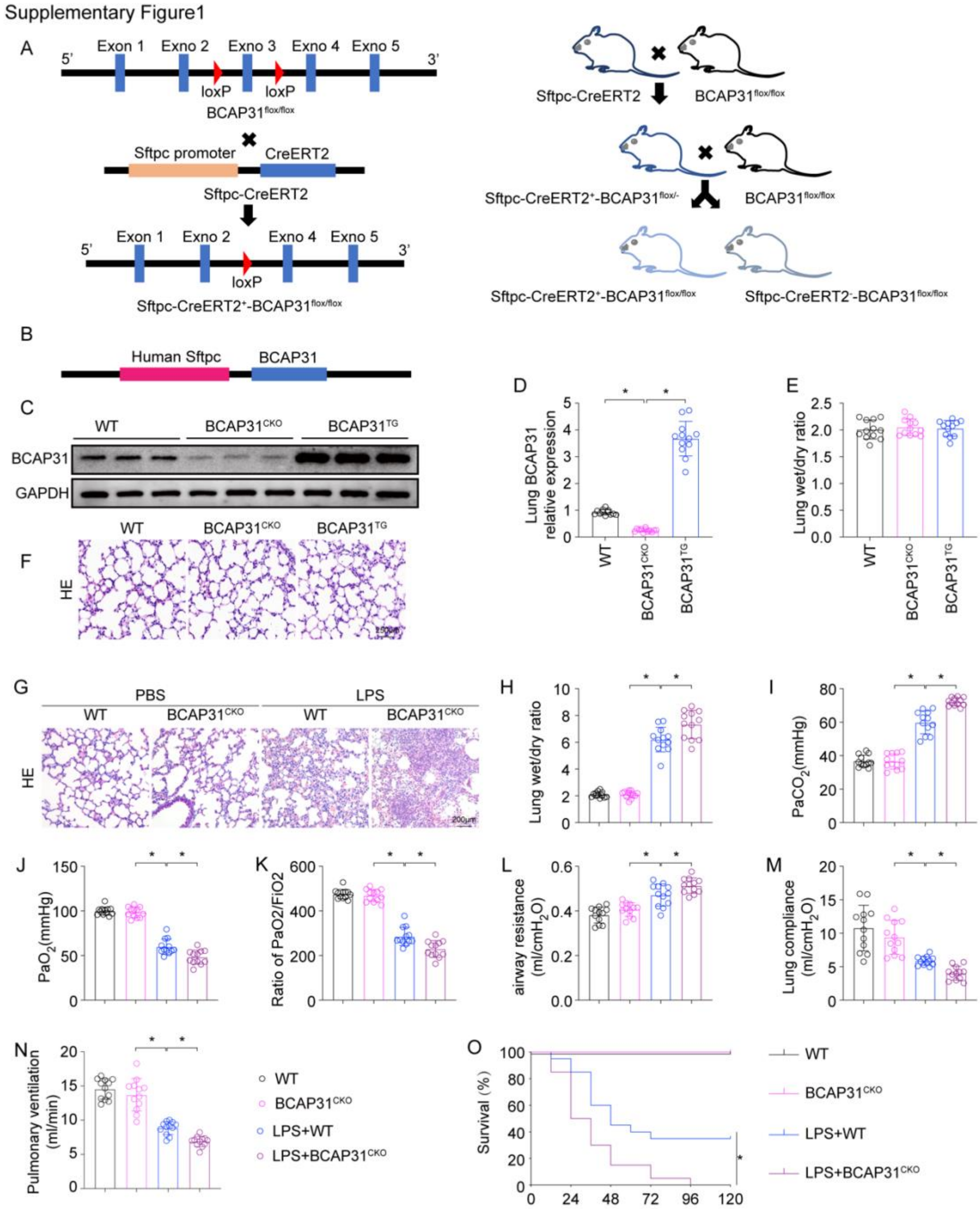

Supplementary Figure 1. BCAP31 knockout aggravates LPS-induce ALI. (n=12/group). (A) Diagram indicating the locations of BCAP31 exons, loxP sites and deletion of exon 3 by Cre recombinase. (B) SFTPC-BCAP31 transgenic mice ($BCAP31^{TG}$) were created by insertion of the full-length genomic coding region of the mouse gene into a transgenic targeting cassette containing the mouse SFTPC gene 5′flanking region. (C-D) BCAP31 levels were assessed in lung tissue via western blot. (E) Lung weight coefficient was measured by a proportion of lung wet weight to total body weight. (F) Representative histological variation of lung obtained from mice of different groups. (G) HE staining images of the lung tissues. (H) Lung wet weight/dry (W/D) weight ratio. (I-K) Blood from the abdominal aorta of mice was extracted for

blood gas analysis. (L-N) Respiratory function, including airway resistance, lung compliance, and pulmonary ventilation was detected by Buxco. ()) the percent survival rate was expressed as a Kaplan-Meier survival curve (n=20 per group). Data are mean ± SD. *P<0.05.

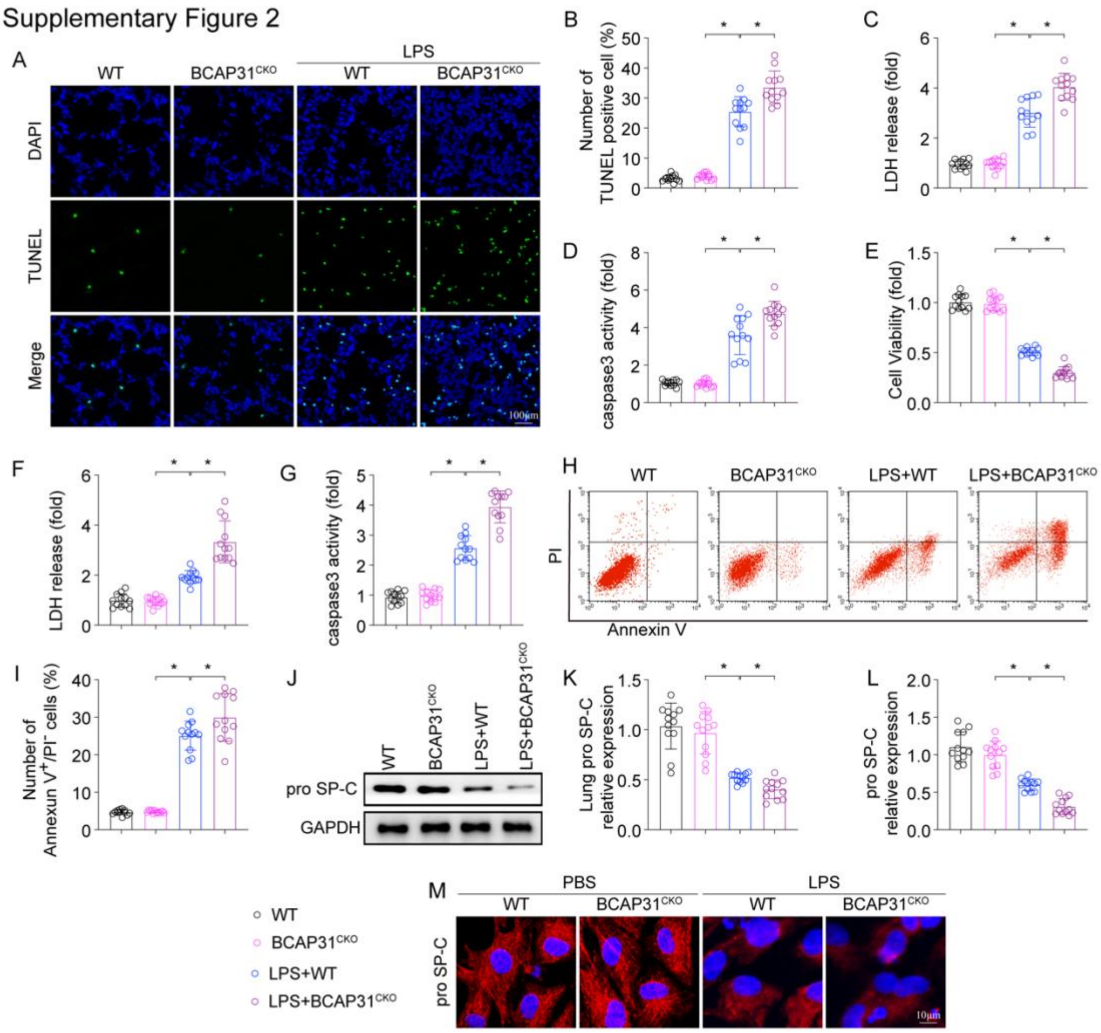

Supplementary Figure 2. Loss of BCAP31 promotes AECII damage followed LPS challenge. (n=12/group). (A-B) TUNEL assay was performed to assess cellular apoptosis in lung tissue. LDH release (C) and caspase-3 activity (D) were detected via ELISA. (E) The cellular viability was detected via MTT assay. (F) LDH release. (G) Caspase3 activity. (H-I) Proportion of apoptotic cells was measured by flow cytometry with Annexin V/PI staining. (J-K) The expression of pro SP-C was measured via western blotting. (L-M) The expression of pro SP-C in AECII cells were measured via immunofluorescence staining. Data are mean ± SD. *P<0.05.

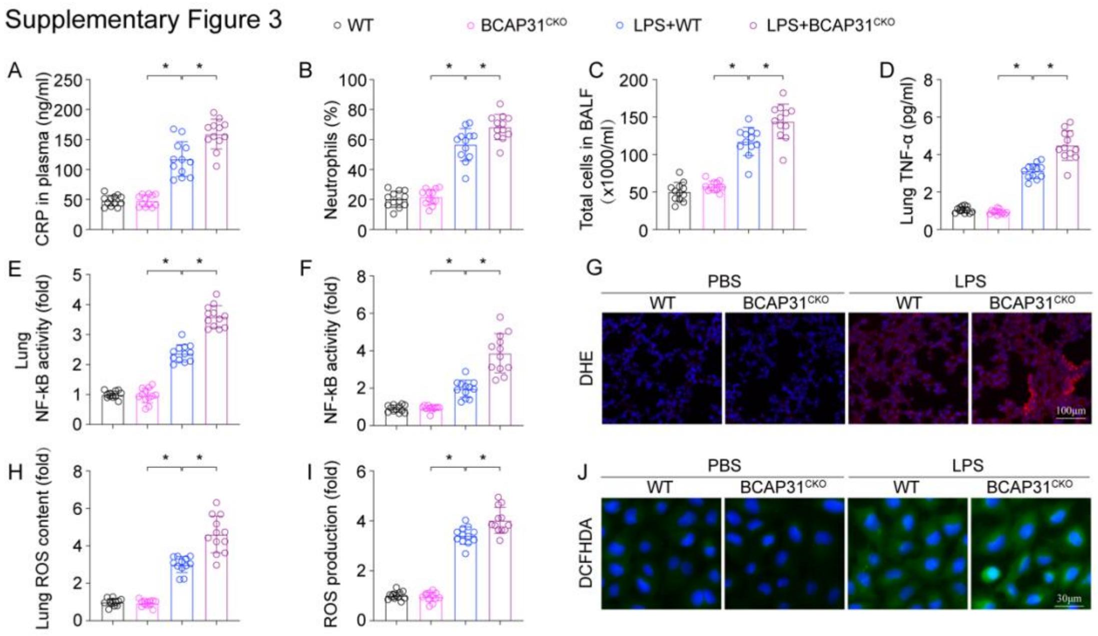

Supplementary Figure 3. BCAP31 deficiency exacerbates inflammatory responses and oxidative stress in ALI (n=12/group). (A) CRP in plasma. (B) NEU (%) in blood. (C) The number of total cells in BALF. (D) The concentrations of TNF-α in lung tissue. (E) The the activity of NF-κB was performed via ELISA. (F) The activity of NF-κB in AECII cells. (G-H) The ROS content in lung tissue. (I-J) DCFHDA was conducted to evaluate the ROS change in AECII cells. Data are mean ± SD. *$P<0.05$.
